# Mutations in the *embB* Gene and Their Association with Ethambutol Resistance in Multidrug-Resistant *Mycobacterium tuberculosis* Clinical Isolates from Poland

**DOI:** 10.1155/2013/167954

**Published:** 2013-12-11

**Authors:** Zofia Bakuła, Agnieszka Napiórkowska, Jacek Bielecki, Ewa Augustynowicz-Kopeć, Zofia Zwolska, Tomasz Jagielski

**Affiliations:** ^1^Department of Applied Microbiology, Institute of Microbiology, Faculty of Biology, University of Warsaw, I. Miecznikowa 1, 02-096 Warsaw, Poland; ^2^Department of Microbiology, National Tuberculosis and Lung Diseases Research Institute, Płocka 26, 01-138 Warsaw, Poland

## Abstract

Ethambutol (EMB) continues to be used as part of a standard drug regimen for the treatment of tuberculosis (TB). Mutations in the *embB* gene and those within its conserved EMB resistance determining region (ERDR) in particular have repeatedly been associated with resistance to EMB in *Mycobacterium tuberculosis*. The aim of this study was to examine the mutational “hot spots” in the *embB* gene, including the ERDR, among multidrug-resistant (MDR) *M. tuberculosis* clinical isolates and to find a possible association between *embB* mutations and resistance to EMB. An 863-bp fragment of the *embB* gene was sequenced in 17 EMB-resistant and 33 EMB-susceptible MDR-TB isolates. In total, eight *embB* mutation types were detected in 6 distinct codons of 27 (54%) *M. tuberculosis* isolates. Mutations in codon 306 were most common, found in both EMB-resistant (9) and EMB-susceptible (11) isolates. Only mutations in codons 406 and 507 were found exclusively in four and one EMB-resistant isolates, respectively. Sequence analysis of the ERDR in the *embB* gene is not sufficient for rapid detection of EMB resistance, and the codon 306 mutations are not good predictive markers of resistance to EMB.

## 1. Introduction

One of the greatest challenges in the fight against tuberculosis (TB) has been the emergence and spread of drug-resistant (DR) and multidrug-resistant (MDR) strains of *Mycobacterium tuberculosis*. The development of new molecular techniques targeting specific molecular mutations associated with drug resistance creates a valuable adjunct to conventional drug susceptibility testing (DST) for *M. tuberculosis*. These techniques can be performed directly on clinical samples without a culturing step and thus allowing a reliable diagnosis of drug-resistant TB to be achieved as fast as within a 24-hour period.

Ethambutol (EMB), an arabinose analogue, is a bacteriostatic, antimycobacterial drug, which has been used for the treatment of TB since the mid-1960s. The drug is routinely recommended for the intensive phase of TB therapy, as part of a four-drug regimen, including isoniazid (INH), rifampicin (RMP), and pyrazinamide (PZA) [1]. Disturbingly, almost 4% of all *M. tuberculosis* clinical isolates have been shown to display resistance to EMB [2].

Ethambutol appears to target the cell wall of tubercle bacilli through interfering with arabinosyl transferases, encoded by the *embCAB* operon, comprised of three homologous genes, designated *embC, embA*, and *embB*, and involved in the biosynthesis of arabinogalactan and lipoarabinomannan, the key structural components of the mycobacterial cell wall. The proposed scenario of EMB action on *M. tuberculosis* is that upon interaction with the EmbCAB proteins EMB inhibits the arabinan synthesis leading to a lack of arabinan receptors for mycolic acids and accumulation of mycolic acids results in cell death [3]. Resistance to EMB has repeatedly been associated with alterations in the *embB *gene, particularly in *embB *codon 306, referred to as EMB resistance determining region (ERDR). Sequence analysis of the ERDR has been considered a rapid screening tool for detection of resistance to EMB [4–6]. Several allelic exchange studies have demonstrated that mutations in codons *embB*306*, embB*406, and *embB*497 are responsible for low and moderate levels of EMB resistance [7, 8]. However, this correlation is uncertain because all these codons have also been found mutated in isolates susceptible to EMB [9–12].

The aim of this study was to examine mutational “hot spots” in the *embB *gene, including the ERDR region, among MDR *M. tuberculosis *clinical isolates from Poland and to find a possible association between *embB* mutations and resistance to EMB.

Part of the results of this study was presented as a poster (A-527-0001-03736) at the 5th Congress of European Microbiologists (FEMS 2013), Leipzig, Germany, July 21–25, 2013.

## 2. Material and Methods

### 2.1. Strains and Drug Susceptibility Testing

A total of 50 *M. tuberculosis *strains isolated from 46 unrelated adult patients (40 men and 6 women; age range: 31 to 79 years; median age: 50.5 years) with pulmonary MDR-TB were included in this study. The isolates were collected at the National Tuberculosis and Lung Diseases Institute in Warsaw during the 3rd national survey on DR-TB throughout 2004 and represented all MDR-TB cases in Poland in that year [13]. Primary isolation, culturing, and species identification of the isolates were done according to standard mycobacteriological procedures, described elsewhere [14]. Resistance determination for first-line anti-TB drugs was performed by using the proportion method on Löwenstein-Jensen (LJ) medium [14]. The critical concentration used for EMB was 2 *μ*g/mL. The *M. tuberculosis* H37Rv reference strain served as a quality control for EMB susceptibility testing.

### 2.2. DNA Extraction and Amplification

Genomic DNA was extracted from *M. tuberculosis* cultures on LJ slants by using the cetyl-trimethyl ammonium bromide (CTAB) method [15]. For EMB resistance, a 863-bp fragment of the* embB* gene was PCR-amplified, with the oligonucleotide primers embBF (5′-CGACGCCGTGGTGATATTCG-3′) and embBR (5′-CCACGCTGGGAATTCGCTTG-3′) and directly sequenced. Amplification reactions were performed in a final volume of 50 *μ*L containing 1x TopTaq buffer PCR, 1.25 U of TopTaq DNA polymerase (Qiagen), 0.2 *μ*M of each primer, 200 *μ*M of each dNTP and 10 ng of DNA template. After initial denaturation at 94°C for 3 min, the reaction mixture was run through 30 cycles of denaturation at 94°C for 30 s, annealing at 58°C for 30 s, and extension at 72°C for 50 s, followed by a final extension at 72°C for 5 min. Amplified fragments were separated by electrophoresis at 3.5 V/cm in 1% agarose gels in 0.5x TBE buffer and visualized by staining with ethidium bromide (0.5 *μ*g/mL) and exposure to UV light (*λ* = 320 nm).

### 2.3. Amplicon Sequence Analysis

Purified PCR amplicons (Clean-Up, A&A Biotechnology) were sequenced by using the BigDye ver. 3.1 Terminator Cycle Sequencing Kit (Applied Biosystems) in the ABI 3130xl Genetic Analyzer (Applied Biosystems). Sequencing was done in both directions using the same forward and reverse primers as those used in the PCR. Sequence data were assembled and analysed with the ChromasPro (ver. 1.7.1) software (Technelysium). The presence of mutations was determined by comparing the obtained sequences with the *M. tuberculosis* reference strain H37Rv sequence of *embB *from GenBank database (http://www.ncbi.nlm.nih.gov/genbank/) using the BLASTN algorithm (http://blast.ncbi.nlm.nih.gov/).

### 2.4. Nucleotide Sequence Accession Numbers

The sequences with novel mutations were deposited in GenBank under the following accession numbers: KF694753 (Met306Ile, Arg507Gly), KF694754 (Leu413Pro), and KF694755 (Glu504Gln).

## 3. Results

Of the 50 MDR-TB isolates under the study, 17 (34%) were resistant to EMB, as measured by the proportion method.

In total, eight *embB* mutation types were detected in 6 distinct codons of 27 (54%) *M. tuberculosis* isolates tested. Thirteen (76.5%) EMB-resistant isolates and 14 (42.4%) EMB-susceptible isolates carried mutations in the analyzed *embB* region. An amino acid change at codon 306 was the most frequent and occurred in 20 (40%) isolates (i.e., in 9/17 EMB-resistant and in 11/33 EMB-susceptible isolates). The Met306Val substitution resulting from an A→G transition at nucleotide position 916 was detected in 4 EMB-resistant and 3 EMB-susceptible isolates, while the Met306Ile substitution, due to either a G→A transition or a G→C transversion at nucleotide position 918, was detected in 5 resistant and 8 susceptible isolates, respectively. The second most common amino acid change was Gly406Ala caused by a transition G→C at nucleotide position 1217. This alteration was found exclusively in 4 EMB-resistant isolates. Only one isolate (EMB-resistant) had a double mutation in the analysed region: G→A at position 918 (Met306Ile) and A→G at position 1519 (Arg507Gly). Other point mutations were identified only in EMB-susceptible isolates and were as follows: T→C at position 1238 (Leu413Pro), A→G at position 1490 (Gln497Arg), and G→C at position 1510 (Glu504Gln). A detailed summary of the sequencing results is provided in Table 1.

## 4. Discussion

Although EMB has been used for the treatment of TB for over 40 years, the molecular mechanisms of EMB resistance still remain poorly understood. Previous studies have correlated the EMB resistance phenotype with mutations in genes of the *embCAB* operon, most notably in the *embB *gene. Mutations at codon position 306 of the *embB* gene have been found to occur most frequently. The high detection rates of mutations at codon *embB*306 among EMB-resistant *M. tuberculosis* isolates were reported from Korea (47%) [16], China (55%) [17], Cuba and the Dominican Republic (70%) [12] and also from countries neighboring to Poland, such as Russia (48%) [18] and Germany (68%) [5]. The role of the *embB*306 alterations in creating resistance of tubercle bacilli to EMB was confirmed by allelic exchange mutagenesis [19]. Additionally, strains with the Met306Ile substitution were found to have lower MICs of EMB (20 *μ*g/mL) than strains with Met306Val or Met306Leu replacements (40 *μ*g/mL) [20]. However, a number of reports have indicated that only less than 35% of EMB-resistant *M. tuberculosis *isolates harbored mutations in the *embB *codon 306 [21, 22]. Moreover, mutations in codon *embB*306 have been found also in *M. tuberculosis* strains susceptible to EMB, and the frequencies of those mutations in EMB-susceptible strains approached those among EMB-resistant strains [9, 11].

The results of this study are in line with previous findings, showing mutations in *embB*306 codon to predominate (40% of all MDR-TB isolates) and to occur at higher frequency in EMB-resistant than EMB-susceptible isolates (53% versus 33%).

Studies on EMB resistance showed that mutations in the *embBAC *gene cluster, outside *embB *codon 306, do occur but are quite rare. Only two other substitutions, found in* embB* codons 406 and 497, have been consistently associated with EMB resistance. The percentage of *embB*406 mutations among EMB-resistant isolates is rather low, usually not exceeding 10%, whereas mutations in codon *embB*497 are twice as frequent [12, 17, 21].

Allelic exchange experiments performed at codons 406 and 497 of the *embB* gene have concluded that point mutations at these codons only slightly increase resistance to EMB [7]. Interestingly, mutations in codons 406 and 497 have—similarly to mutations in codon 306—been identified also in *M. tuberculosis* strains susceptible to EMB [17, 23]. In our study mutations at *embB*406 were found exclusively in 4 (23%) EMB-resistant strains, whereas a single mutation at *embB*497 was found only in an EMB-susceptible isolate. Mutations at codons other than 306, 406, and 497 were identified only in two EMB-susceptible and one EMB-resistant isolates.

Three novel mutations in the examined fragment of the *embB* gene were observed in this study. The sequence variations in codons 504 and 507 have already been described before in EMB-resistant isolates, yet the amino acid replacements were different from those observed here [24]. The substitution at codon 413 is reported for the first time. Of the three new mutations described in this work, only that in codon 507 may have an impact on EMB resistance, since it was found in an EMB-resistant isolate. Yet, the extent of this impact was masked by the cooccurrence of the *emb306* change in that isolate.

More than a half (54%) of MDR-TB clinical isolates tested had mutations in the examined region of the *embB *gene. These mutations occurred nearly twice as frequently in EMB-resistant than EMB-susceptible isolates (76.5% versus 42%).

The high frequency of *embB* mutations with no association between the presence of mutation and EMB-resistant phenotype can be explained by the fact that mutations in the *embB* gene occur significantly more frequently in MDR than EMB-monoresistant strains [9, 25, 26]. Several studies have demonstrated a strong association between *embB*306 mutations and resistance to INH or RMP, or MDR phenotype [19, 25, 26]. It has been suggested that *embB*306 mutations may have selective advantage upon treatment with multiple drugs. In other words, these mutations inhibit the synergistic effect of anti-TB drugs when used in combination [19]. The molecular mechanism behind this phenomenon can only be speculated and may involve changes in the cell wall permeability as a result of *embB*306 mutations [19].

Another possible explanation for the lack of correlation between the *embB* gene alterations and EMB resistance may relate to a cumulative effect of multiple mutations on the development of EMB resistance. Acquisition of resistance to EMB is thought to be a gradual process that may involve numerous genes [3, 27]. Strains bearing *embB* mutations are susceptible to EMB because these mutations alone are not sufficient to generate EMB resistance unless accompanied by alterations in other genetic loci. Recently, Safi et al. have shown that mutations in the *embB, embC*, *Rv3806c*, and *Rv3792* genes, involved in the decaprenylphosphoryl-*β*-D-arabinose (DPA) biosynthetic and utilization pathway, produce a wide range of ethambutol MICs by interacting in different ways and that the acquisition of EMB resistance does not occur in a single step but requires a multistep process [28].

Finally, conclusions concerning EMB resistance can be inaccurate because of the false-negative DST results, and thus importance of mutations in the *embB *gene can be underestimated. Quite often, the MIC values for EMB have varied depending upon culture medium, strain condition, or the DST method used [29]. The results of previous studies have shown that EMB resistance can indeed be phenotypically missed by routine laboratory procedures [30, 31].

## 5. Conclusions

Despite the limitations of the study in terms of size and time frame of the sample, our results confirm previous observations that sequencing of the ERDR within the *embB* gene is not sufficient for rapid detection of EMB resistance and that the codon 306 mutations are not good markers for the prediction of resistance to EMB. Analysis of other genetic loci is needed for the identification of more specific mutations associated with EMB resistance.

## Figures and Tables

**Table 1:** Mutations detected in 50 MDR *M. tuberculosis* isolates under the study.

Mutation^a^		No. (%) of isolates with detected mutation	
Nucleotide	Amino acid	EMB-resistant (*n* = 17)	EMB-susceptible (*n* = 33)
A → G (916)	Met → Val (306)	4 (23.5)	3 (9.1)
G → A (918)	Met → Ile (306)	3 (17.6)	5 (15.1)
G → C (918)	Met → Ile (306)	1 (5.9)	3 (9.1)
G → C (1217)	Gly → Ala (406)	4 (23.5)	—
T → C (1238)*	Leu → Pro (413)	—	1 (3)
A → G (1490)	Gln → Arg (497)	—	1 (3)
G → C (1510)*	Glu → Gln (504)	—	1 (3)
G → A (918) and A → G (1519)*	Met → Ile (306) and Arg → Gly (507)	1 (5.9)	—

^a^Numbers in brackets indicate nucleotide positions and amino acid residue positions; *novel mutation.
